# Assessing the Quality and Reliability of AI-Generated Responses to Common Hypertension Queries

**DOI:** 10.7759/cureus.66041

**Published:** 2024-08-02

**Authors:** Aleena Vinufrancis, Hussein Al Hussein, Heena V Patel, Afshan Nizami, Aditya Singh, Bianca Nunez, Aiah Mounir Abdel-Aal

**Affiliations:** 1 Internal Medicine, Apollo Hospitals, Thrissur, IND; 2 Internal Medicine, Hamad Medical Corporation, Doha, QAT; 3 Internal Medicine, Gujarat Cancer Society (GCS) Medical College, Hospital, and Research Center, Ahmedabad, IND; 4 Medicine and Surgery, Appollo Medical College, Hyderabad, IND; 5 Cardiology, Bhartiya Vidyapreet Medical College and Hospital, Sangli, IND; 6 Internal Medicine, Universidad autonoma e Guadalajara, Guadalajara , MEX; 7 Pediatrics, Facult of medicine , University of Alexandria, Egypt, EGY

**Keywords:** patient counseling, chatsonic, chatgpt, hypertension, healthcare, artificial intelligence

## Abstract

Introduction: The integration of artificial intelligence (AI) in healthcare, particularly through language models like ChatGPT and ChatSonic, has gained substantial attention. This article explores the utilization of these AI models to address patient queries related to hypertension, emphasizing their potential to enhance health literacy and disease understanding. The study aims to compare the quality and reliability of responses generated by ChatGPT and ChatSonic in addressing common patient queries about hypertension and evaluate these AI models using the Global Quality Scale (GQS) and the Modified DISCERN scale.

Methods: A virtual cross-sectional observational study was conducted over one month, starting in October 2023. Ten common patient queries regarding hypertension were presented to ChatGPT (https://chat.openai.com/) and ChatSonic (https://writesonic.com/chat), and the responses were recorded. Two internal medicine physicians assessed the responses using the GQS and the Modified DISCERN scale. Statistical analysis included Cohen’s Kappa values for inter-rater agreement.

Results: The study evaluated responses from ChatGPT and ChatSonic for 10 patient queries. Assessors observed variations in the quality and reliability assessments between the two AI models. Cohen’s Kappa values indicated minimal agreement between the evaluators for both the GQS and Modified DISCERN scale.

Conclusions: This study highlights the variations in the assessment of responses generated by ChatGPT and ChatSonic for hypertension-related queries. The findings highlight the need for ongoing monitoring and fact-checking of AI-generated responses.

## Introduction

The advent of artificial intelligence (AI) has taken the world by storm and rightly so. Large language models like ChatGPT and ChatSonic have a tremendous ability to carry out difficult tasks quite rapidly and easily. Its use in medical science is being extensively researched in both clinical and academic settings [1]. It is only a matter of time until it proves to be the structural framework of applied health sciences in the future. It uses a massive data set to generate conversational and easy-to-understand responses [2].

Hypertension is a widely prevalent chronic disease characterized by a rise in systolic and/or diastolic blood pressure. The exact definition varies based on different guidelines but the most widely accepted cut-off for diagnosis of hypertension is systolic pressure ≥140 mmHg and diastolic ≥90 mmHg [3]. It is associated with various long-term morbidities including strokes and myocardial infarctions [4]. Maintaining blood pressure at the optimum levels constantly is the key to preventing these long-term morbidities and mortalities associated with the disease.

The persistent nature of hypertension makes it a difficult disease to manage for both the patients and the doctors. Patients will have everlasting questions regarding the disease. In a time where doctors have limited time dedicated to each patient, the use of large language models like ChatGPT can be used effectively to counsel the patient to improve health literacy and understanding of the disease [5]. It will significantly lessen the burden on doctors while also providing reliable and repeatable responses to patients. This study was conducted to assess the quality and reliability of responses generated by ChatGPT and ChatSonic in addressing common patient queries about hypertension.

## Materials and methods

A virtual cross-sectional observational study was conducted over a one-month period commencing in October 2023. The study evaluated the responses generated by large language models, specifically ChatGPT and ChatSonic, in addressing patient queries related to hypertension. Ethics committee approval was not required as the study did not involve human participants or direct patient interaction.

The study was conducted entirely in a virtual environment, utilizing the online platforms where ChatGPT (https://chat.openai.com/) and ChatSonic (https://writesonic.com/chat) operate. The study duration encompassed October 2023, during which data collection and assessments took place. The primary outcomes of interest were the quality and reliability of responses generated by ChatGPT and ChatSonic. The exposures involved patient queries related to hypertension and no potential confounders were identified for this study.

The authors of this study devised 10 common patient queries about hypertension. The questions were derived from typical inquiries an individual with hypertension may have regarding the condition. The questions were as follows: 1 - What is hypertension; 2 - What causes hypertension; 3 - Is hypertension life-threatening?; 4 - Is hypertension curable?; 5 - Can I cure my hypertension with diet and exercise? 6 - How often should I get my blood pressure checked? 7 - What should I do if I forget to take my medications? 8 - If my blood pressure is controlled, can I stop the medication?; 9 - What are the expected side effects from hypertensive medications?; and 10 - Can I live a good life with hypertension? On October 31, 2023, the queries were presented to two AI software, ChatGPT (https://chat.openai.com/) and ChatSonic (https://writesonic.com/chat), and the generated responses were recorded in Microsoft Word documents. The blinded responses were then sent to two evaluators (Evaluator 1 and Evaluator 2). The selected evaluators had expertise in internal medicine and could independently assess the responses, ensuring a comprehensive evaluation and reducing bias.

Each evaluator assessed the blinded generated responses based on the Global Quality Scale (GQS) (Table 1) and the Modified DISCERN scale (Table 2) for both quality and reliability. The overall quality of the generated response was evaluated utilizing the GQS, employing a 5-point Likert scale ranging from 1 to 5, with a score of 5 indicating excellent response quality [6]. DISCERN serves as a tool intended to assist consumers of health information in evaluating the quality of information regarding treatment options [7]. The validity of the generated response was evaluated using a modified version of the DISCERN scale, in which higher scores corresponded to increased reliability. In this modified scale, each “no” response to a question is assigned a score of 0, while each “yes” response is assigned a score of 1 [7]. 

**Table 1:** Global quality score

Score	Global score description
1	Poor quality, poor flow of the site, most information missing, not at all useful for patients
2	Generally poor quality and poor flow, some information listed but many important topics missing, of very limited use to patients
3	Moderate quality, suboptimal flow, some important information is adequately discussed but others poorly discussed, somewhat useful for patients
4	Good quality and generally good flow, most of the relevant information is listed, but some topics not covered, useful for patient
5	Excellent quality and excellent flow, very useful for patients

**Table 2:** Modified DISCERN score Score 1 for every Yes; Score 0 for every No. Total score is calculated (the highest score is 5, and the lowest score is 0)

Item	Questions
1	Are the aims clear and achieved?
2	Are reliable sources of information used? (i.e., publication cited, the responses are from valid studies/sources)
3	Is the information presented balanced and unbiased?
4	Are additional sources of information listed for patient reference?
5	Does it refer to areas of uncertainty?

The study data, comprising a sample size of 10, was systematically organized in a Microsoft Excel spreadsheet. Statistical analyses were conducted using StataCorp. 2023, Release 18 (StataCorp LLC., College Station, TX) software. Inter-rater agreement between the two evaluators for the modified DISCERN and GQS scores was evaluated using Cohen’s Kappa value. The interpretation of Cohen’s Kappa values is as follows: a score of > 0.8 denotes “near perfect agreement,” 0.61-0.8 signifies “substantial agreement,” 0.41-0.6 indicates “moderate agreement,” 0.21-0.4 represents “fair agreement,” and < 0.2 reflects “slight agreement.” A score of 1 is interpreted as “perfect agreement” while a score of 0 signifies “no agreement between the two raters.” The predetermined significance level for this analysis was set at p < 0.05.

Key findings, including modified DISCERN and GQS scores, were tabulated to provide a succinct overview of the evaluation process. Graphical representations, such as bar charts or scatter plots, were employed where applicable to visually depict trends or variations in the data.

## Results

Ten frequently asked questions from patients regarding hypertension were chosen for evaluation. These queries were directed to two AI platforms, namely ChatGPT and ChatSonic. Subsequently, the responses generated were evaluated by two assessors using the GQS and DISCERN scales, which gauge quality and reliability, respectively. The ensuing outcomes are illustrated in the forthcoming tables and figures.

**Figure 1:**
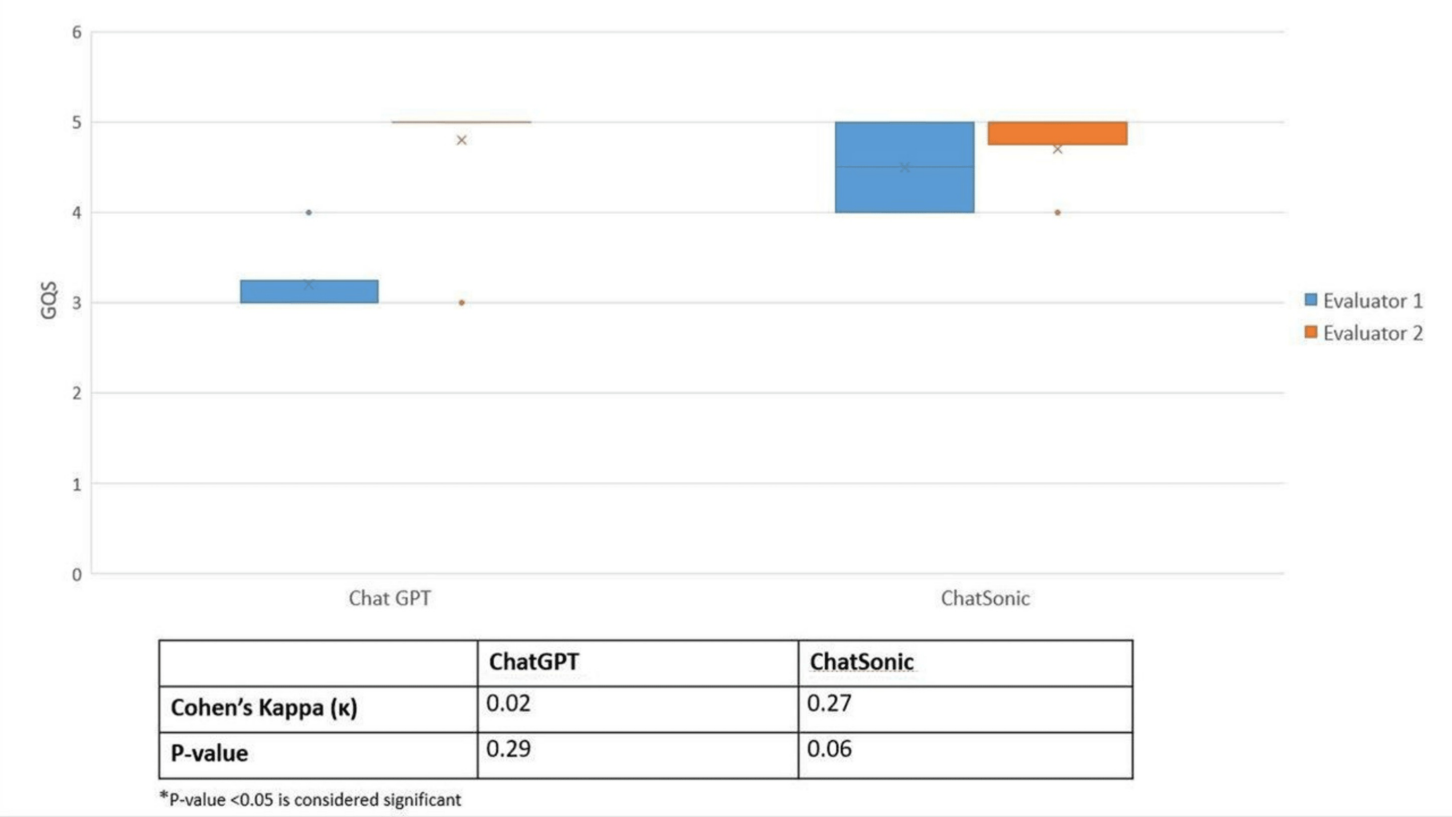
Average GQS given to ChatGPT and ChatSonic generated responses. GQS - Global Quality Scale

Figure 1 demonstrates the evaluation of ChatGPT responses by two assessors. Evaluator 1 identified high and very high-quality responses (GQS = 4 & 5) in two (20%), out of 10 instances, while Evaluator 2 observed this quality in nine (90%) out of 10 responses. Regarding reliability, as assessed using the DISCERN scale, Evaluator 1 found high and very high reliability (DISCERN = 4 & 5) in three (30%) out of 10 instances, whereas Evaluator 2 noted this reliability in all 10 (100%) out of 10 responses. The figure illustrates variations in assessments between the two evaluators and provides an overview of ChatGPT's response quality and reliability.

**Figure 2:**
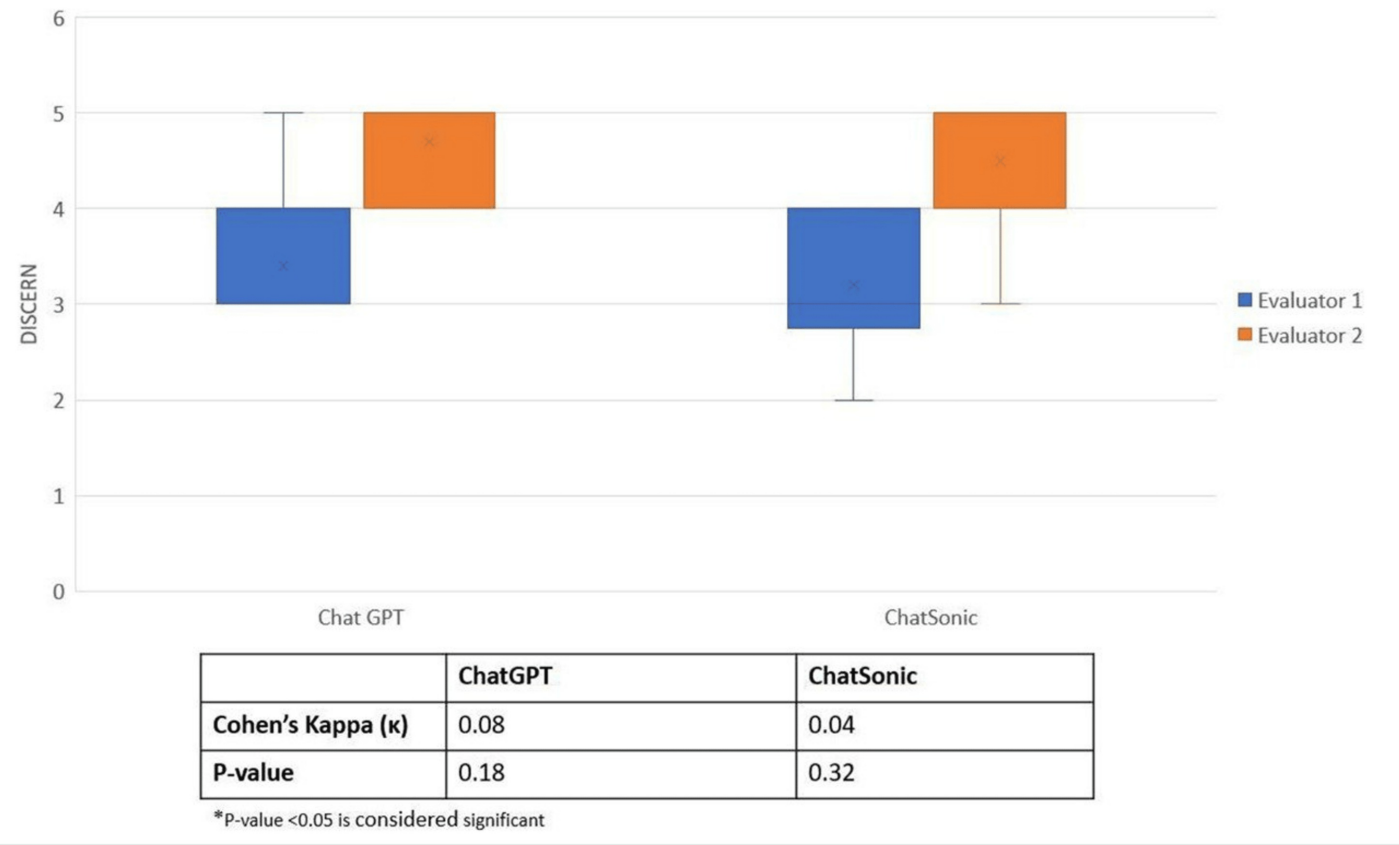
Average DISCERN given to ChatGPT and ChatSonic generated responses.

Figure 2 illustrates the assessment of ChatSonic responses by two evaluators. Evaluator 1 assigned high and very high-quality ratings (GQS= 4 & 5) to all 10 (100%) of the 10 responses, while Evaluator 2 observed this quality in nine (90%) out of 10 responses. Furthermore, in terms of reliability, as evaluated by the DISCERN scale, Evaluator 1 identified high and very high reliability (DISCERN = 4 & 5) in four (40%) out of 10 instances, while Evaluator 2 noted this level of reliability in nine (90%) out of 10 responses. The figure provides insights into the quality and reliability of ChatSonic's responses based on the evaluations of the two assessors.

GQS scores assigned by the two evaluators for ChatGPT-generated responses exhibit very weak agreement (k = 0.02), with no statistically significant difference observed (p = 0.29 > 0.05). Conversely, for ChatSonic-generated responses, there is a fair agreement (k = 0.27) in quality (GQS) scores between the two evaluators, although the associated p-value of 0.06 is considered somewhat borderline. In both cases, for ChatGPT and ChatSonic, the kappa values are notably low, indicating minimal agreement between the two evaluators (Figure 1).

For ChatGPT, a slight agreement beyond chance is seen, although it lacks statistical significance. Similarly, with ChatSonic, there is only a slight agreement beyond chance, and the associated p-value is greater than 0.05, indicating a lack of statistical significance. These findings suggest that the level of agreement between the two evaluators is not robust, and the observed agreement may be attributed to random chance (Figure 2).

## Discussion

Several factors piqued our interest in performing this study. The paramount reason is the increasing dependence on AI software by patients for healthcare-related queries. As more and more people depend on this software to make critical decisions about their health, evaluation of these responses is becoming the need of the day. There have been studies where the responses generated by AI software were preferred in terms of quality and empathy to the responses given by physicians [8]. Research also shows that the existing version of ChatGPT narrates data from existing internet literature in a general manner and that’s where the importance of fact-checking of the responses generated by them comes into play [9]. We hope that if properly monitored this software could help physicians in responding to patient queries in the future.

In this study, two evaluators who are experts in the field of managing systemic hypertension assessed the quality and reliability of responses generated by ChatGPT and ChatSonic regarding patient queries for hypertension. The evaluation of AI-driven responses to common patient queries on hypertension using ChatGPT and ChatSonic yielded varied outcomes. GQS scoring of the responses generated by ChatGPT and ChatSonic showed very weak and fair agreement between the two evaluators respectively. In the DISCERN scoring, it was noticed that the responses generated by ChatGPT and ChatSonic showed a slight agreement between the evaluators.

A GQS was used to determine the quality of the responses generated by the AI software [6,10]. GQS scoring of responses generated by both ChatGPT and the evaluators showed a kappa value of 0.2 indicating very weak agreement between them and it was not statistically significant. Whereas responses generated by ChatSonic had a kappa value of 0.27 indicating fair agreement between the two evaluators.

The reliability of the information generated by the AI software was evaluated by Quality Criteria for Consumer Health Information (DISCERN) [11,12]. The Discern grading of the responses showed a kappa value of 0.08 and 0.04 indicating that the level of agreement between the two evaluators is not very strong and the observed agreement may be due to chance.

A study has found that the text-based chatbot designed for hypertension self-management demonstrated favorable usability, with participants successfully completing tasks and expressing a need for features to enhance the user experience [13]. Similarly, amidst the COVID-19 pandemic, various commercial services, including Microsoft Azure and Amazon Web Service, introduced integrated frameworks for symptom checkers and other medical content providing a valuable foundation for the creation and advancement of future chatbots [14]. It is noteworthy that while the positive aspects of AI in healthcare are evident, the lack of recognition for free-text responses in health information could present potential patient safety risks without the implementation of proper measures and human oversight [15].

Limitations

Several limitations merit consideration. The study focused on two specific AI models, limiting the generalizability of findings to other platforms. The use of only two evaluators may introduce bias, and a larger evaluator pool could enhance the study's robustness. Additionally, the study evaluated responses to a specific set of queries, which may not capture the full spectrum of AI performance in addressing diverse patient concerns. Generalizing findings to broader AI applications in healthcare requires caution. Future research should encompass a more extensive range of AI models, involve diverse evaluators, and assess responses to a broader array of patient queries to enhance the generalizability of conclusions.

## Conclusions

This study focuses on the application of AI-driven models, specifically ChatGPT and ChatSonic, in addressing patient queries related to hypertension. Despite variations in evaluator assessments, both models demonstrated the potential to deliver high-quality responses, thus enhancing health literacy and patient understanding of chronic diseases. Moving forward, efforts to enhance the generalizability of findings should include a broader range of AI models, involve diverse evaluators, and assess responses to a wider array of patient queries. Specialized chatbots designed for medical science to respond to patient queries can be developed and trained using verified information from reputable sources such as the World Health Organization (WHO). These chatbots should be promoted and made freely accessible for patients to use. However, the responses generated by these chatbots must undergo thorough scrutiny by medical professionals and government agencies to ensure accuracy and reliability.
